# Exploring the medication duration based on the effect of traditional Chinese medicine on postoperative stage I-III colorectal patients: a retrospective cohort study

**DOI:** 10.18632/oncotarget.14567

**Published:** 2017-01-09

**Authors:** Qi Shi, Shanshan Liu, Wen Li, Shaoqi Zong, Susu Han, Wei Yang, Hongjia Li, Fenggang Hou

**Affiliations:** ^1^ Oncology Department of Municipal Hospital of Traditional Chinese Medicine, Shanghai University of Traditional Chinese Medicine, Shanghai 200071, China

**Keywords:** colorectal cancer, medication duration, traditional Chinese medicine

## Abstract

**Purpose:**

To clarify the effect of tradional Chinese medicine (TCM) on different stage patients and to explore medication duration based on survival analysis.

**Results:**

523 and 294 patients were respectively in the TCM group and the control group. For all patients, 6-year disease-free survival (DFS) was 57.6% after TCM and 46.6% after non-TCM (*p* = 0.0006). 6-year DFS for patients with stage I disease in the TCM group was 79.5% compared with 89.1% in the control group (*p* = 0.65). For patients with stage II disease, 6-year DFS was 63.1% in the TCM group compared with 50.2% in the control group (*p* = 0.054), and for patients with stage III disease, it was 43.3% in the TCM group compared with 22.0% in the control group (*p* = 0.0000).

**Materials and Methods:**

Data for patients with stage I-III disease between 2004 and 2013 were retrieved for this study, who underwent TCM after surgery were in the TCM group and the others were in the control group. Clinic appointments or phone were used to collect data by research assistants. Survival data were collected on Nov 2015 from the database, which is continuously updated by the researchers.

**Conclusions:**

TCM is associated with significantly improved disease-free survival, in particular for patients with stage III disease. Among of these, TCM is not necessary for patients with stage I disease, and postoperative patients with stage II disease should be recommended to take 2 years of TCM. For patients with stage III disease, adherence to medication of TCM during the 6-year follow-up is worthy of being recommended.

## INTRODUCTION

Colorectal cancer (CRC) is one of the most common malignant tumors [1–3]. Surgery is still the main treatment of CRC, but approximately 35% of patients develop to recurrence [4]. Traditional Chinese medicine (TCM) play an important role in adjuvant therapy. Two study summarized that TCM had three advantages to prevent and treat tumor: preventing tumorigenesis; attenuating toxicity and enhancing the treatment effect; and reducing risk of recurrence and metastasis [5, 6]. In previous studies, we found TCM could reduce the risk of metastatic recurrence of patients with CRC, and prolong the disease-free survival (DFS) [7–9], which was in agreement with other studies [10–13]. However, whether TCM can all reduce the recurrence risk for patients with different stage disease is still unknown. In addition, treatment duration of TCM is too long, and this is one of the main reasons for patients to interrupt the treatment. How long should patients receive TCM treatment?.

Therefore, we undertook this study in order to clarify the effect of TCM on patients with different stage disease and to provide evidence on the patients’ medication duration.

## RESULTS

### Baseline characteristics

817 patients were included in this cohort study. Table 1 shows the baseline and tumor characteristics. In the TCM group, 461 (88.1%) of 523 patients with adenocarcinoma compared with 236 of 294 (80.3%) in the control group. (*p* = 0.01). In addition, difference of histodifferentiation between two groups was significant (*p* = 0.01). 420 (80.3%) of 523 patients received adjuvant chemotherapy in the TCM group and 204 (69.4%) of 294 patients in the control group (*p* = 0.000). The baseline characteristics after propensity score matching was shown in Supplementary Table 1. Meanwhile, results of multiple factor analysis were shown in Supplementary Table 2. Among of them, hazard ratio (HR) of TCM was 1.67 and 95% CI was 1.33 to 2.11. Follow-up in the TCM group with a median of 55.6 months compared with 40.8 months in the control group (*p* = 0.16).

**Table 1:** Baseline and tumor characteristics of patients before propensity score matching

	TCM group (*n* = 523)	Control group (*n* = 294)	*p* value
age (y)			
< 60	199	97	0.15
≥ 60	324	197	
gender			
male	274	161	0.51
female	249	133	
location			
colon	312	179	0.73
rectum	211	115	
pathology			
adenocarcinoma	461	236	0.01*
non-adenocarcinoma	4	2	
unknown	58	56	
histodifferentiation			
poorly	52	29	0.01*
moderately	322	149	
well	17	11	
known	132	105	
TNM stage			
I	85	52	0.36
II	219	134	
III	219	108	
chemotherapy			
yes	420	204	0.00*
no	103	90	
radiotherapy			
yes	38	17	0.42
no	485	277	
comorbidities			
yes	270	131	0.05
no	253	163	

*statistical difference.

### Effect of TCM on all stage I-III CRC patients

**Figure 1:**
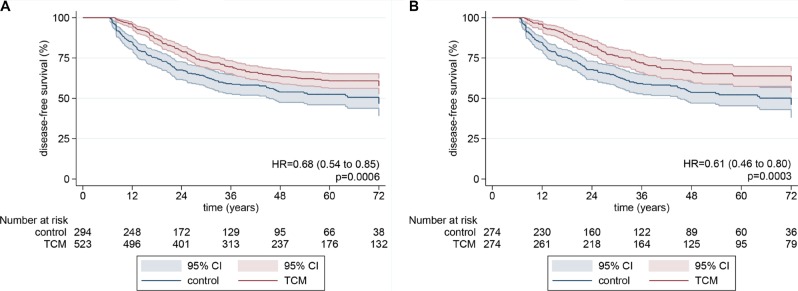
Kaplan-Meier disease-free survival curves for all patients *P* values from log-rank tests. Pointwise confidence bands are 95% CIs. (**A**) before propensity scoring; (**B**) after propensity scoring.

1–6 year disease-free survival in the TCM group were 95.6%, 79.0%, 69.6%, 63.6%, 60.9% and 57.6%. Those in the control group were 84.6%, 67.1%, 58.7%, 53.9%, 52.5% and 46.6% (Supplementary Figure 1). In Figure 1, the result of median disease-free survival (mDFS) in the control group was 64.4 months and that in the TCM group was not attached (HR:0.68; log-rank *p* = 0.0006). After propensity score matching, disease-free survival was also significantly improved after TCM with non-TCM (HR: 0.74; log-rank *p* = 0.02), although mDFS were not attached in both TCM and control group.

**Figure 2:**
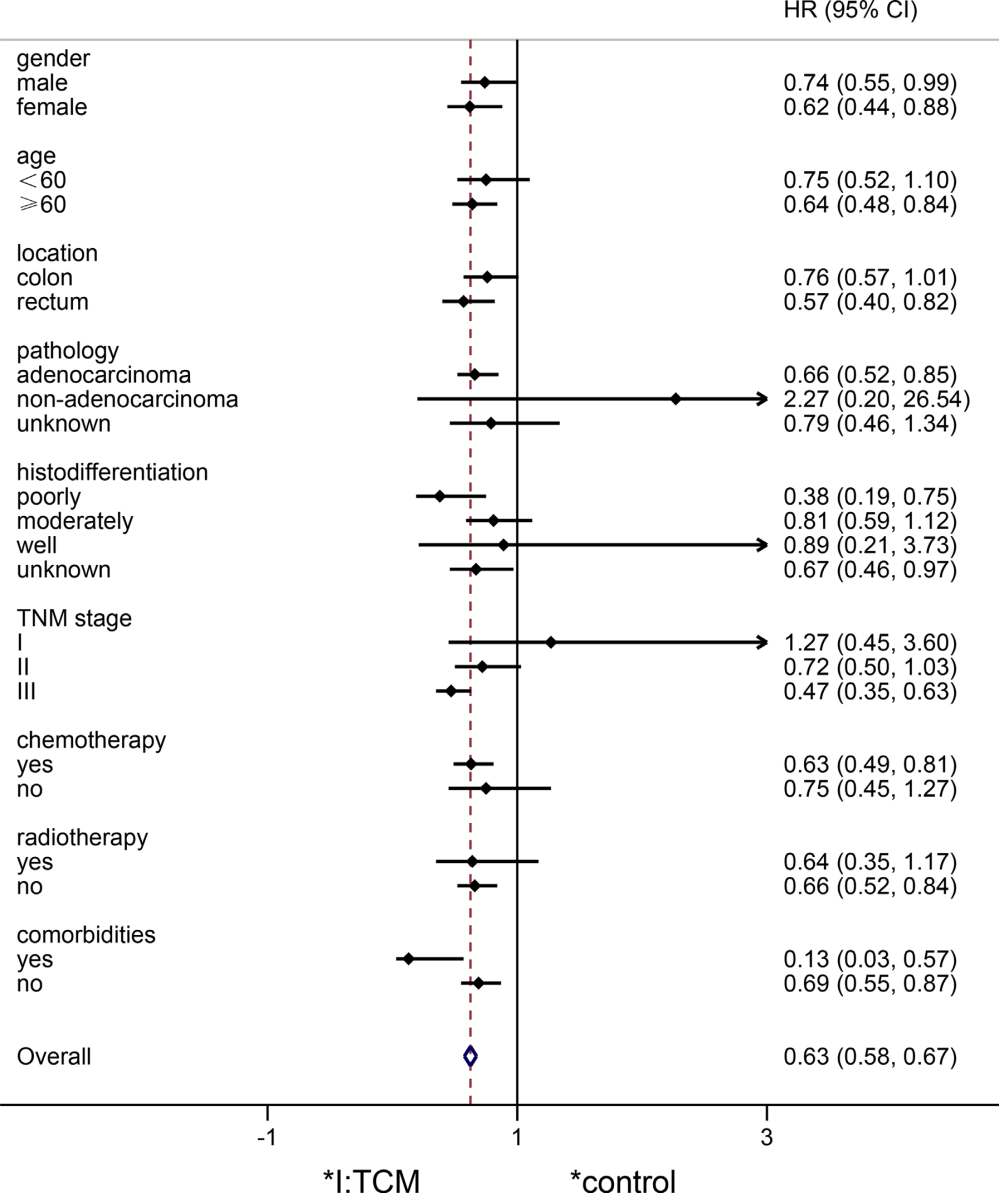
Forest plot of hazard ratio between the TCM group and the control group 95% CI did not cross the equivalent linear x = 1 means log-rank *p* < 0.05; whereas, log-rank *p* > 0.05.

In addition, HRs in different subgroups were analyzed in Figure 2. HRs of the TCM group were general lower than those of the control group (*HR* = 0.63, 0.58 to 0.67). Among of these, risk of patients in the TCM group were significantly lower than those in the control group in terms of female, no less than 60 years old, rectal cancer, adenocarcinoma, poorly differentiated, stage III disease, receiving chemotherapy, no radiotherapy and no comorbidities (95% confidence interval cross the equivalent linear x = 1).

### Effect of TCM on different stage CRC patients

**Figure 3:**
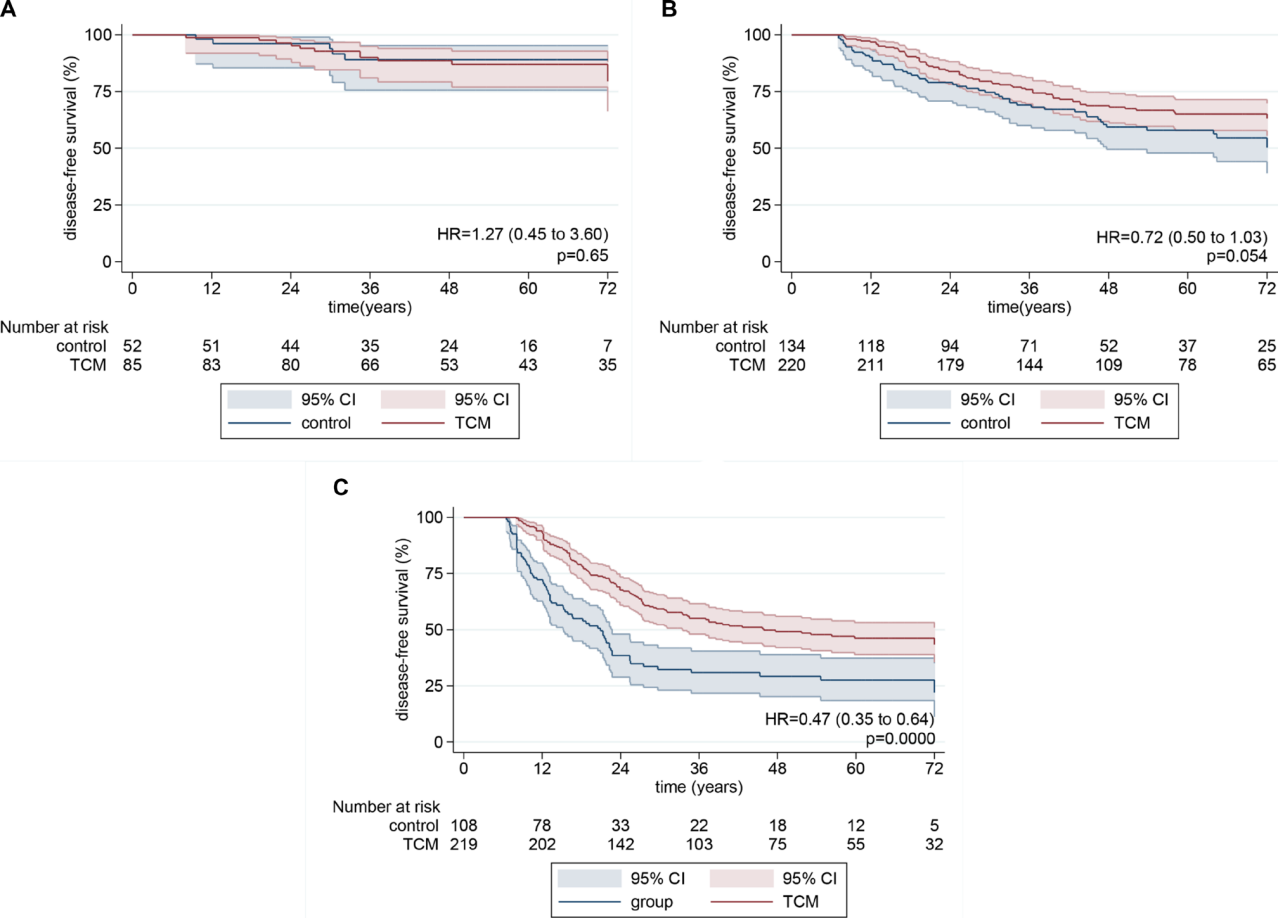
Kaplan-Meier disease-free survival curves for patients with different stage disease (**A**) Stage I. (**B**) Stage II. (**C**) Stage III. *P* values from log-rank tests. Pointwise confidence bands are 95% CIs.

6-year disease-free survival for 137 patients with stage I disease was 79.5% in the TCM group and 89.1% in the control group (*HR* = 1.27, log-rank *p* = 0.65; Figure 3A). For 353 patients with stage II disease, 6-year disease-free survival was 63.1% in the TCM group compared with 50.2% in the control group (*HR* = 0.72, log-rank *p* = 0.054; Figure 3B). For 327 patients with stage III disease, 6-year disease-free survival was 43.3% in the TCM group compared with 22.0% in the control group (*HR* = 0.47, log-rank *p* = 0.0000; Figure 3C).

## DISCUSSION

Our data indicate that TCM is independently associated with significantly improved disease-free survival, in particular for patients with stage III disease.

Recently, several studies have proved that TCM can effectively prevent the metastatic recurrence of CRC. A clinical study from Longhua Hospital compared 37 cases in integrated treatment group with 41 cases in western medicine group, and their results showed that TCM treatment is important to improve the outcome of stage II or III CRC in elderly patients [14]. Another two trials from Zhou LY and Xue JZ both indicated that TCM combined chemotherapy could prevent relapse and benefit survival [15, 16]. Likewise, in terms of DFS, our study proved the significant effect of Chinese herbs on I-III stages patients. However, it is not clear whether the effect of TCM differ in characteristics of patients. Our further subgroup analysis reveal that TCM has a tendency to further reduce the risk of metastatic recurrence in elderly (no less than 60 years old) female patients, and has the same tendency in patients with rectal cancer, adenocarcinoma, poorly differentiation, stage III disease, comorbidities, or chemotherapy. We consider that may be related to the role of TCM in efficacy enhancing and toxicity reducing, improving immunity and quality of life [17–24].

For patients with stage II-III disease, several studies indicated that adjuvant chemotherapy was necessary for those with stage III disease, but not for all patients with stage II disease, only those at high-risk were more likely to benefit from adjuvant chemotherapy [25–28]. Meanwhile, for patients with stage I disease, adjuvant therapy was not required for them, and they only needed to receive regular colonoscopy. However, it was not clear whether TCM was effective for all patients with stage I-III disease. Our data indicated that TCM had a significant effect on patients with stage III disease, only a tendency on those with stage II disease, and for stage I disease the effect hadn't be discovered. The only other study, which compared 159 in the TCM group and 59 in the control group, reported a similar improved outcome for stage III, but not for patients with stage II disease [29]. We consider it is very possible that the inhibition rate of TCM in lymph node is higher than that in primary tumor, such as 5-FU, VP-16, THP and MMC [30].

### Strengths and limitations

This is an objective, large sample, research of different effect on I–III stage CRC. Through this research, TCM can be individually used for CRC patients with stage I–III disease, especially for those stage III patients. Meanwhile, one limitation is also in our study. Role of adjuvant therapy for patients with high-risk stage II CRC still remains contention [31–34]. Given margin status, local perforation and bowel obstruction were not sufficient, we thus did not analyse the effect of TCM on stage II patients at high risk.

## MATERIALS AND METHODS

### Study design and participants

**Figure 4:**
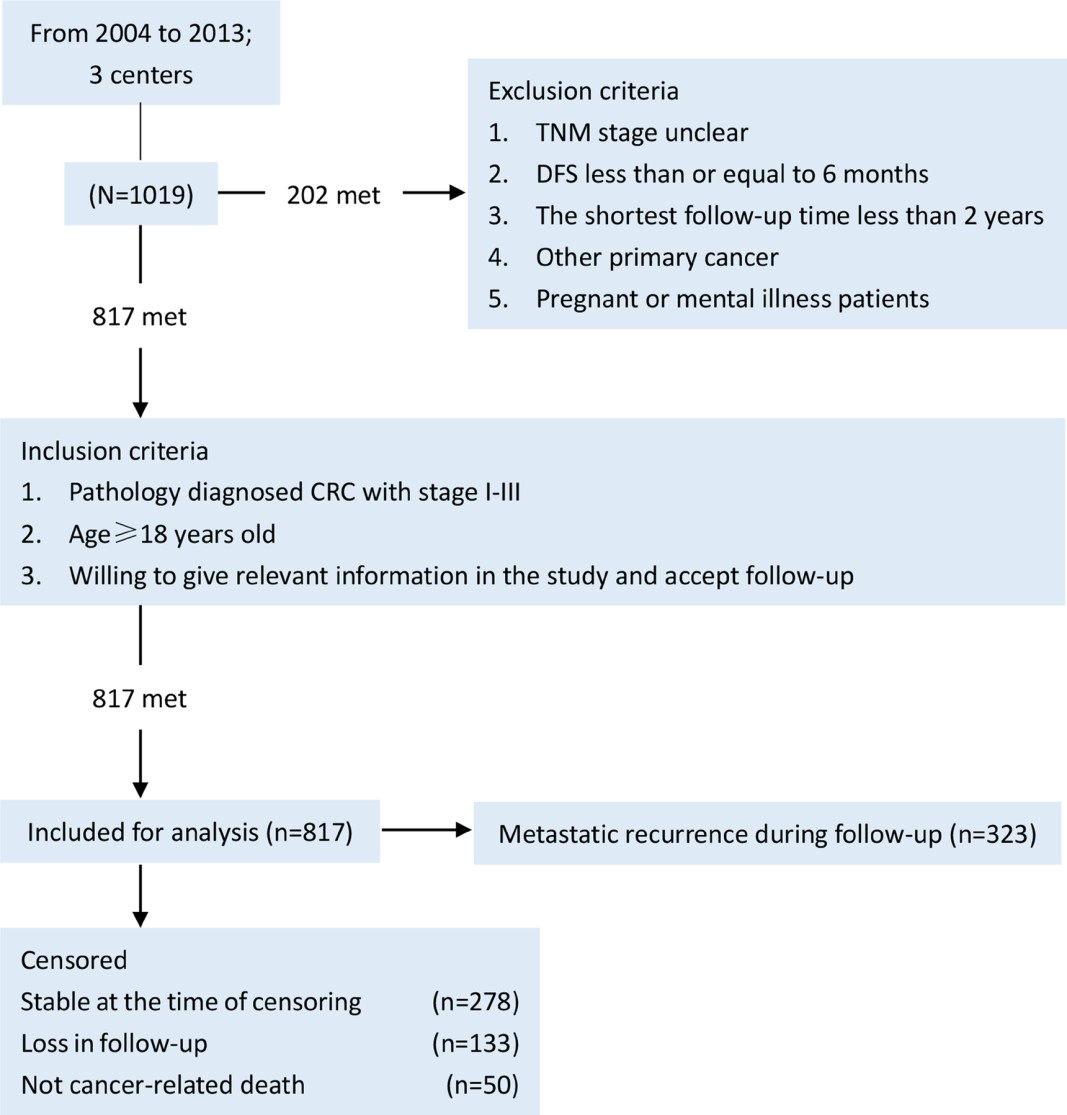
Study patient selection and censoring flow chart CRC, colorectal cancer; DFS, diease-free survival.

The study was a retrospective, multicenter, non-intervention, observational, cohort study involving municipal hospital of traditional Chinese medicine, Shuguang hospital and Yueyang hospital. These three hospitals were affiliated to Shanghai University of traditional Chinese medicine. Research ethics boards from all these three centers approved the study protocol. Between April 2004 and November 2013, 1019 patients with stage I-III CRC were screened and 819 were enrolled onto the study (Figure 4). Of these, 524 patients were systematically treated with systematic TCM (TCM group) and 295 were not (control group). Notably, systematic TCM treatment was defined as continuously medication more than six months before metastatic recurrence.

### TCM treatment

Traditional Chinese herbs were administrated by decoction, which was suggested to take at least 6 months continuously. One cycle of treatment was defined as 2 weeks. Dosage of herbs were decided by the attending physician according to clinical symptoms of patients.

### Data collection

Clinical data were gathered via clinic appointments or phone by research assistants come from three centers. Variables or outcome included gender, age, location, pathologic type, histodifferentiation, TNM stage, chemotherapy, radiotherapy, comorbidities, prescriptions of TCM, and status of recurrence or metastasis. Although patients were observed for different amounts of time, data were collected every 6 months postoperatively. The maximum of follow-up was 6 years in order to avoid the possibility of loss in follow-up.

### Statistical analyses

Categorical and continuous variables were respectively analysed by Pearson's *χ^²^* test and Student *t* tests. Multiple factor analysis was performed by Cox regression. DFS was measured from the date of diagnosis to the date of metastatic recurrence, and was evaluated with Kaplan-Meier curves and log-rank tests for categorical data. Propensity scores were created with logistic regression modeling the probability of a patient undergoing TCM treatment on gender, age, location, pathologic type, histodifferentiation, TNM stage, chemotherapy, radiotherapy and comorbidities. A 1:1 match with random matching order and 0.1 caliper was done, replacement was not allowed. All statistical analyses were performed using SPSS (version 22.0) and Stata (version 12.0). Hypothesis testing was two sided and conducted at the 5% level of significance.

## CONCLUSIONS

Above all, our study prompt that TCM can generally improve disease-free survival of CRC patients with stage I–III. However, TCM is not necessary for patients with stage I disease, and postoperative patients with stage II disease should be recommended to take 2 years of TCM because most relapses occur within 2 years after surgery [35, 36]. For patients with stage III disease, adherence to medication of TCM during the 6-year follow-up [37] is worthy of being recommended.

## SUPPLEMENTARY FIGURE AND TABLES


