# Supplementary material for: Exploring the medication duration based on the effect of traditional Chinese medicine on postoperative stage I-III colorectal patients: a retrospective cohort study
Source: Oncotarget. 2017 Jan 9;8(8):13488–95. doi: 10.18632/oncotarget.14567 (PMC5355114; doi:10.18632/oncotarget.14567)
Supplement: Supplementary file 1 [file oncotarget-08-13488-s001.pdf]

Exploring the medication duration based on the effect of traditional Chinese medicine on postoperative stage I-III colorectal patients: a retrospective cohort study

Supplementary Materials

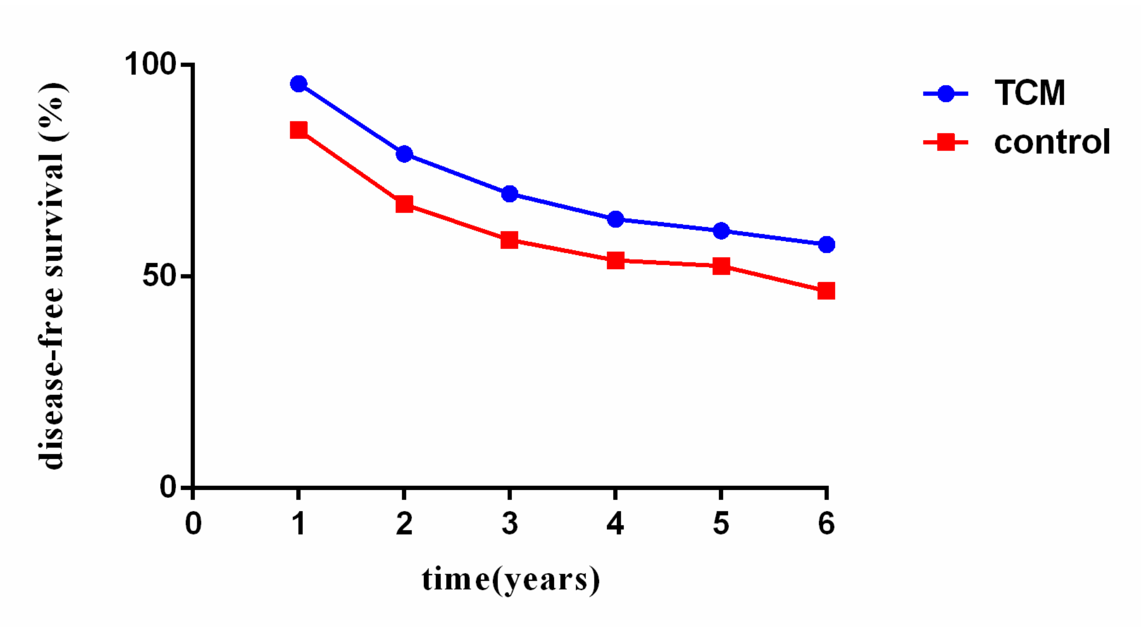

Supplementary Figure 1: 1–6 year disease-free survival in the TCM and control group.

Supplementary Table 1: Baseline and tumor characteristics of patients after propensity score matching

	**TCM group (*n* = 274)**	**Control group (*n* = 274)**	***p* value**
age (y)			
< 60	90	90	1.00
≥ 60	184	184	
gender			
male	146	145	0.93
female	128	129	
location			
colon	165	166	0.93
rectum	109	108	
pathology			
adenocarcinoma	229	229	
non-adenocarcinoma	1	2	0.84
unknown	44	43	
histodifferentiation			
poorly	26	28	
moderately	150	146	0.94
well	13	11	
known	85	89	
TNM stage			
I	49	46	
II	126	124	0.89
III	99	104	
chemotherapy			
yes	186	193	0.52
no	88	81	
radiotherapy			
yes	17	15	0.72
no	257	259	
comorbidities			
yes	134	126	0.49
no	140	148	

Supplementary Table 2: Results of multiple factor analysis through cox regression

Factor	HR	95% CI	*P*
age	0.73	0.58–0.91	0.01*
gender	1.11	0.86–1.42	0.42
location	0.79	0.63–1.01	0.06*
pathology			
non-AC vs AC	1.32	0.40–4.28	0.65
unknown vs AC	1.01	0.69–1.47	0.96
histodifferentiation			
moderately vs poorly	0.86	0.58–1.25	0.42
well vs poorly	1.13	0.51–2.48	0.77
unknown vs poorly	1.53	0.99–2.36	0.05
TNM stage			
II vs I	2.74	1.65–4.57	0.00*
III vs I	6.48	3.90–10.74	0.00*
chemotherapy	1.24	0.91–1.71	0.18
radiotherapy	2.84	2.03–3.97	0.00*
TCM	1.67	1.33–2.11	0.00*
comorbidities	1.03	0.81–1.30	0.82

Abbreviations: HR, hazard ration; CI, confidence interval; AC, adenocarcinoma; TCM traditional Chinese medicine; *statistical difference.
